# Discriminability of selected periods III‒IV elements in photon-counting computed tomography using a pixelated CdTe detector

**DOI:** 10.1038/s41598-025-26812-5

**Published:** 2025-11-28

**Authors:** Kengo Shibuya, Shunya Nakasone, Taiki Yoshii, Akira Yunoki, Hirotaka Sakai, Hiromi Kimura, Takeshi Fujiwara, Takeru Takeuchi, Jun Kawarabayashi

**Affiliations:** 1 Regulatory Standard and Research Department, Nuclear Regulation Authority, Roppongi 1-9-9, Minato-ku, Tokyo, 106-8450 Japan; 2 National Metrology Institute of Japan, National Institute of Advanced Industrial Science and Technology, Umezono 1-1-1, Tsukuba, Ibaraki 305-8568 Japan; 3 Faculty of Science and Engineering, Tokyo City University, Tamazutsumi 1-28-1, Setagaya-ku, Tokyo, 158-8557 Japan

**Keywords:** Elemental discrimination, Periods III and IV elements, Photon-counting computed tomography (PCCT), Pixelated CdTe detector, Nuclear backend, Bhattacharyya coefficient, Imaging techniques, Nuclear waste, Imaging techniques

## Abstract

This study introduces a new approach in photon-counting computed tomography (CT) for elemental discrimination in periods III and IV of the periodic table of elements, which have not been covered by conventional methods that rely on the K-absorption edge of the target element. The present method features a pixelated CdTe detector with positional sensitivity and energy resolution. The authors changed its energy threshold level to determine the worthiness of counting an energy deposition by an X-ray photon in a pixel rather than changing the tube voltage in accordance with the target element as in the conventional method. The differences between the images are visualised on a scatter plot against various threshold levels, which clearly separates iron (atomic number 26) and copper (atomic number 29) with minimal overlap after normalising the CT values of aluminium and air to 0 and −1000, respectively. The discriminability was quantified using the Bhattacharyya coefficient. This elemental discrimination method does not rely on the K-absorption edge of the target element and can therefore be broadly applied to other elements in non-destructive (non-invasive) industrial and biomedical CT inspections.

**Supplementary Information:**

The online version contains supplementary material available at 10.1038/s41598-025-26812-5.

## Introduction

Elemental discrimination in photon-counting computed tomography (CT) has been actively investigated in medical imaging to distinguish contrast agents from biological tissues such as bone. Contrast agents typically contain heavy elements, such as iodine, barium, and gadolinium, which are strong X-ray absorbers in periods V and VI of the periodic table of elements^1–7^. Conversely, this study focuses on lighter elements, such as aluminium, iron, and copper, which belong to periods III and IV and are representative metals found in radioactive waste from nuclear power plants^8^. Discrimination of light elements is more challenging owing to the limited applicability of the K-edge imaging method, which has been successfully applied to heavy elements^1,4,5,9–11^. The K-edge energies of iodine, barium, and gadolinium are 33.17 keV, 33.74 keV and 50.24 keV, respectively^12^. X-ray photons at these energies are efficiently detected by a semiconductor detector, providing a good element-specific contrast between the images acquired above and below the K-edge energy of the target. By contrast, the K-edge energies of aluminium, iron and copper are 1.58 keV, 7.11 keV, and 8.98 keV, respectively^12^. Such low-energy photons are difficult to count using commercially available semiconductor detectors operating at room temperature^13^.

This study proposes a method for photon-counting CT for elemental discrimination in periods III and IV of the periodic table. Our method is characterised by changing the energy threshold of the radiation detector to determine whether an energy deposition of an X-ray photon should be counted as an event, rather than changing a radiation generator parameter, such as the X-ray tube voltage, as in conventional K-edge imaging. The method requires an X-ray detector with both positional sensitivity and energy resolution. Position-sensitive semiconductor detectors are well suited to this purpose; we employed a pixelated CdTe detector in this study.

Our motivation stems from the need for clearance and disposal of solid waste from nuclear power plants. The clearance system in many countries permits their recycling and disposal as general waste, provided that the activity concentration (Bq/kg) does not exceed the specified levels corresponding to the dose criteria^14,15^. In Japan, the clearance system was previously restricted to three types of materials: metals, glass wool, and concrete debris. These materials can be packaged almost uniform manner. Following the revision of the regulations in 2020, all solid materials became subject to clearance, and the current system covers heterogeneous waste, such as power distribution panels and cables. In assessing the activity of such non-uniform waste, Yoshii et al. highlighted the need for prior information, such as CT images, to prevent underestimation of the activity when setting a conversion coefficient from a detector’s count rate (s^− 1^) to activity (Bq)^16^. A more accurate correction for self-shielding can be achieved by identifying the constituent elements and using published values for their attenuation coefficients.

Furthermore, there are additional requirements for elemental discrimination in clearance and disposal. Copper in the waste may contain a weak-β-emitter ^63^Ni (half-life 101.2(15) years, maximum β-ray energy 66.945(5) keV, average β-ray energy 17.425(6) keV^17,18^ produced via the ^63^Cu(n, p)^63^Ni reaction^19^. However, ^63^Ni is difficult to measure by non-destructive methods, such as radiation detection^20,21^; therefore, identifying copper (atomic number 29, *Z* = 29) by discriminating it from iron (*Z* = 26) is useful. In underground disposal, detecting aluminium (*Z* = 13) is also crucial, as this metal gradually corrodes and generates hydrogen gas upon contact with cement, an alkaline material. This gas generation poses a long-term risk of explosive incidents during intermediate-depth disposal^22,23^.

This paper introduces a measurement and analysis method for distinguishing three typical metals (aluminium, iron, and copper) in nuclear power plant waste using photon-counting CT and demonstrates its feasibility. These base metals are also important resources in general recycling, and clearance systems contribute to realising a low-carbon society^24–26^. Because the method is independent of the K-edge energy of the target material, it can be broadly applied to other elements in non-destructive (non-invasive) industrial and biomedical CT inspections.

## Materials and methods

### Radiation detector

We used a commercial X-ray detector, a pixelated CdTe semiconductor detector (WidePIX, ADVACAM, Czech Republic)^27–29^, which incorporates a Medipix signal processor^30,31^ developed at CERN. Each pixel size was 55 μm × 55 μm, and 3840 × 256 pixels of 1.0-mm-thick CdTe crystals^32^ were arranged in a gapless rectangular array, yielding a sensitive area of 211 mm × 14 mm. This detector features two energy thresholds and is operated as follows (charge summing mode^27,30^. An energy deposition by an X-ray photon in a pixel above the first threshold level (FTL) triggers the processor to sum the deposited energy in neighbouring pixels. If the summation exceeds the second threshold level (STL), the processor counts the event at the pixel receiving the maximum deposition. In this study, the FTL was fixed at 30 keV, which is higher than the K-edge of copper (8.98 keV). The STL was set at 60 keV, 120 keV, or 180 keV, as summarised in Table 1, to enable comparison under three conditions. The STL plays a crucial role in this method, as discussed later.

**Table 1:** Measurement conditions.

	Tube voltage/kV	FTL/keV	STL/keV	Tube current/mA	Acquisition time/s (1 projection)	Total counts (1 projection)
Condition A	250	30	60	0.5	2.0	1.7 × 10^9^
Condition B			120	3.0	15	1.5 × 10^9^
Condition C			180	6.0	20	1.2 × 10^9^

The primary variation is in the second threshold level (STL), with the other parameters adjusted accordingly.

### Radiation generator

By contrast, unlike in the K-edge imaging, the X-ray spectrum remained unchanged. The X-ray generator used was an MXR 320 (COMET, Switzerland), operated at a fixed tube voltage of 250 kV, which is higher than the voltage typically used in biological imaging due to the higher density of the present subjects^33–35^. As noted above, the tube current was set to 0.5 mA, 3.0 mA or 6.0 mA depending on the STL, considering that the detector’s sensitivity decreases with increasing STL. The acquisition time per projection was 2 s, 15 s, or 20 s, resulting in approximately the same number of counts across the detector: 1.7 × 10^9^, 1.5 × 10^9^, and 1.2 × 10^9^, respectively. To assess the effect of STL on elemental discriminability, the number of counts was approximately equalised to ensure comparable statistical noise in the images. The focal spot size was set to 1.0 mm. A 1.0-mm-thick copper plate positioned at the X-ray port reduced the radiation exposure to the detector circuits by partially filtering the low-energy component of the X-ray spectrum, thereby preventing occasional communication errors with the PC.

### Subjects

**Fig. 1:**
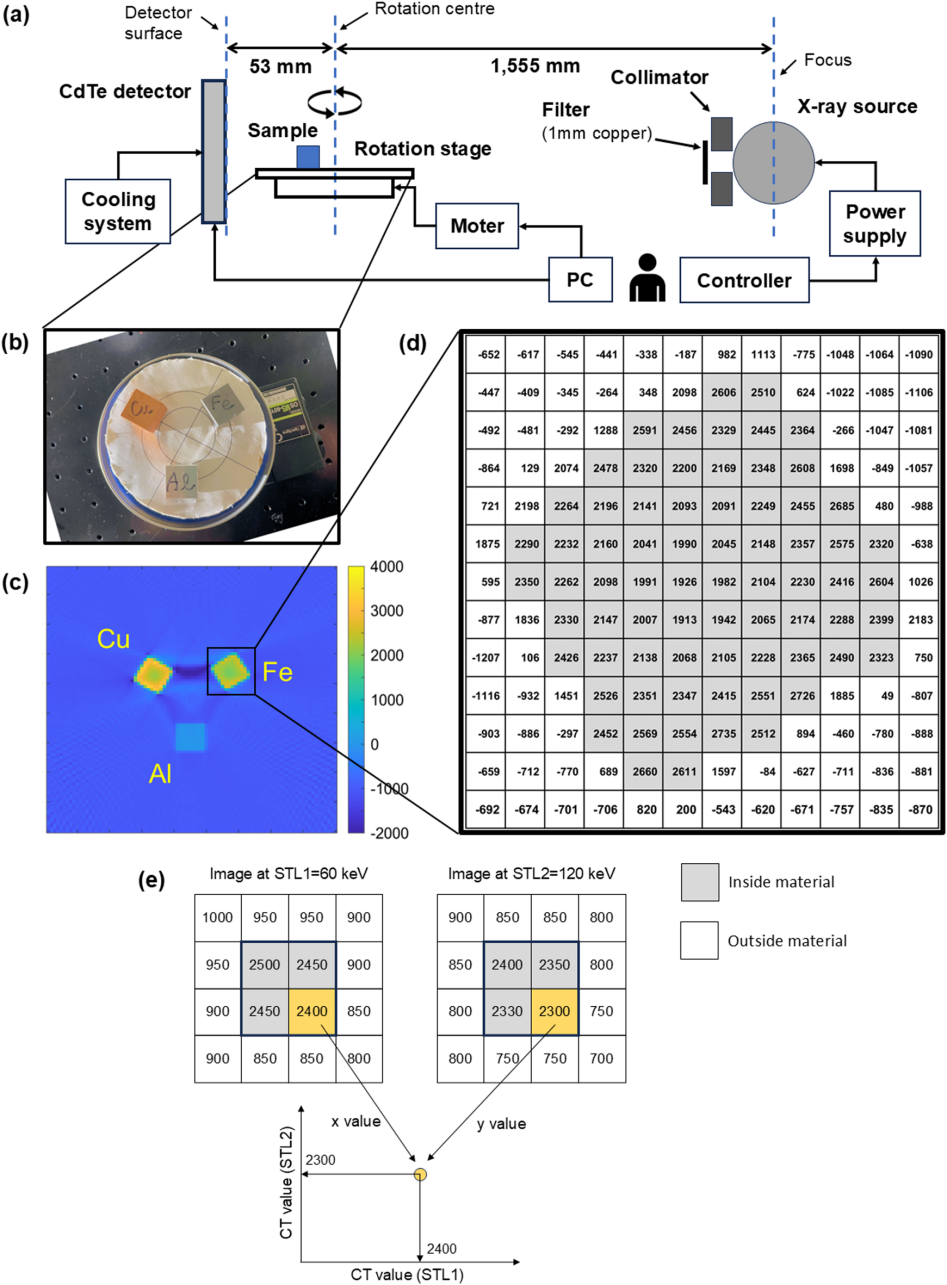
(**a**) Schematic diagram of the experimental setup, showing a CdTe detector and an X-ray source positioned on either side of a sample stage mounted on an auto-rotating table. The distance from the detector surface to the stage rotation centre was 53 mm, and that from the centre to the X-ray source focal spot was 1555 mm. Control signals for applying high voltage to the detector, acquiring data, and regulating the stage motor were transmitted to and received from a laptop PC. The PC and the controller for the X-ray source’s tube voltage and current were operated manually. The detector was equipped with a cooling system that circulates liquid coolant. A 1.0-mm-thick copper plate positioned at the X-ray port reduced radiation exposure of the detector circuits by partially filtering the low-energy component of the X-ray spectrum to prevent occasional communication errors with the PC. Additionally, 5-cm-thick lead collimators were used to reduce unnecessary exposure to the detector. (**b**) Photograph of the subject cubes (copper, upper left; iron, upper right; aluminium, below; each side is 20 mm long) placed on the auto-rotating table. (**c**) Reconstructed image obtained at the STL of 120 keV, with the colour bar indicating the normalised CT values (see Eq. (1) for the normalisation). (**d**) Normalised CT value of each pixel near the iron cube. Grey-hatched pixels are considered inside the material in this study, whereas pixels with CT values between the material and air are treated outside the material. One pixel corresponds to 2.2 mm in length. (**e**) Procedure for constructing a 2D scatter plot (elemental map). For example, there is a 2 × 2 material pixels (hatched) within a 4 × 4 field-of-view pixels, with each pixel showing the normalised CT values obtained at STLs of 60 keV (left) and 120 keV (right). The value of the lower right pixel in the material is 2400 and 2300 in the left and right images, respectively, and is plotted in the elemental map below at coordinates (*X*, *Y*) = (2400, 2300).

Figure 1a illustrates a schematic diagram of the experimental setup. The subjects were three cubes, each 20 mm in length on a side, made of different metals (aluminium, iron, and copper), and placed on an auto-rotating stage, as shown in the photograph (Fig. 1b). The stage was rotated by 2.0° after completing each acquisition in a projection, and 180 acquisitions were taken to obtain 360° data in each condition. Three conditions were measured successively with a total rotation of 360° × 3, as described later. The X-ray generator and detector were fixed on opposite sides of the stage. The distance from the detector surface to the stage rotation centre was 53 mm, and from the centre to the generator focal spot was 1555 mm. These distances allowed us to assume a parallel X-ray beam for the image reconstructions in this paper.

### Image reconstruction and normalisation

The sinogram was generated as follows: (i) All 3840 horizontal pixels and 8 of the 256 vertical pixels were extracted from the detector’s sensitive area of 3840 × 256 to obtain a slice matrix of 3840 × 8 (corresponding to 211 mm × 0.44 mm) for a single acquisition. (ii) The counts in the vertical direction were averaged to produce a compressed slice matrix of 3840 × 1. (iii) The 180 compressed slice matrices (i.e. covering 360°) were then combined to form a sinogram of 3840 × 180. This sinogram was reconstructed into a two-dimensional (2D) image of 150 × 150 pixels, with each pixel representing a length of 2.2 mm, using a standard 2D filtered back-projection (FBP) algorithm^36^.

After image reconstruction, we normalised the CT values such that the average values of pixels assumed to be aluminium and air were 0 and −1000, respectively, using the following equation:

1 $$N(x,y)=1000\frac{n(x,y)-{\bar{n}}_{\text{Al}}}{{\bar{n}}_{\text{Al}}}$$

where *n*(*x*, *y*) and *N*(*x*, *y*) denote the CT values of the pixel at position (*x*, *y*) before and after normalisation, respectively, and $\bar{n}_{\text{Al}}$ denotes the average CT value of pixels assumed to be aluminium before normalisation. Specifically, pixels with CT values intermediate between the material and air are regarded as lying outside the material (cf. Fig. 1d). Equation (1) assumes ${\bar{n}}_{\text{air}}=0$, where $\bar{n}_{\text{air}}$ denotes the average CT value of pixels considered to be air before normalisation. This normalisation is analogous to that used in medical CT, namely, Hounsfield units^4,33^, which normalise water and air pixels (or voxels in 3D) to 0 and −1000, respectively.

### Elemental map

After the above normalisation, we constructed a new scatter plot from the two images obtained under different conditions listed in Table 1. The STL is the most critical parameter in each condition. For example, if two images are selected, one obtained under condition A (STL at 60 keV) and the other under condition B (STL at 120 keV), the scatter plot is constructed as shown in Fig. 1e. When the CT value of a pixel is *P*_1_ in the reconstructed image obtained under condition A, and another CT value of the same pixel is *P*_2_ in the image obtained under condition B, the information of this pixel is plotted at (*X*, *Y*) = (*P*_1_, *P*_2_) in the new 2D scatter plot. In this way, the method projects all pixels within the subjects onto a scatter plot, where the points reveal element-specific distributions. We refer to this scatter plot as an elemental map. As discussed below, the elemental map enables the discrimination of copper from iron, which is not possible using a single measurement condition.

### Metric

Elemental discriminability depends on the degree of overlap between the CT value distributions of the elements. To quantify the overlap, we used the Bhattacharyya coefficient as a metric, defined by Eq. (2a) for one-dimensional (1D) distributions and Eq. (2b) for 2D distributions^37–39^.

2a $${B}_{1}=\sum_{i}\sqrt{{P}_{i}\cdot {Q}_{i}},\quad \sum_{i}{P}_{i}=\sum_{i}{Q}_{i}=1$$

2b $${B}_{2}=\sum_{j}\sum_{i}\sqrt{{P}_{ij}\cdot{Q}_{ij}},\quad \sum_{j}\sum_{i}{P}_{ij}=\sum_{j}\sum_{i}{Q}_{ij}=1$$

The elemental distributions (1D or 2D) were histogrammed using an appropriate number of bins. Two 1D vectors or two 2D matrices, corresponding to the frequencies of the bins, are denoted as $P$ or $Q$, where ${P}_{i}$ and ${Q}_{i}$ represent vector elements, and ${P}_{ij}$ and ${Q}_{ij}$ represent matrix elements. Both frequency distributions are normalised so that the sum of their elements equals 1. The Bhattacharyya coefficient is then calculated by multiplying the vectors or matrices in accordance with Eqs. (2a) or (2b). It takes a maximum value of 1 for two identical distributions, and a minimum value of 0 for two orthogonal distributions with no overlap, i.e. $0\le {B}_{1}\le 1$ and $0\le {B}_{2}\le 1$. Examples of calculation using actual data are provided in Supplementary Materials [see Equations (S1)‒(S5) for the 1D case and Equations (S6)‒(S10) for the 2D case].

This metric has been employed in many fields to assess similarity, such as product recommendations on digital marketing platforms^37^, pattern matching in computer vision^38^, and protection schemes in transmission line diagnostics^39^. These applications commonly use the robustness of the Bhattacharyya-based approach to sparse or noisy data. Such robustness is advantageous for the present CT data analysis, which contains cupping noise due to beam hardening, where CT values do not follow a normal distribution. Furthermore, because the 1D and 2D calculations are fundamentally based on the same procedure, this metric provides an appropriate framework for evaluating the superiority of the 2D map in this study.

To the best of our knowledge, no universally accepted threshold for the Bhattacharyya coefficient has been established to indicate minimal overlap, as its appropriate value is context-dependent, including considerations such as analytical conservatism. In many disciplines, a confidence interval spanning two standard deviations (2σ) is commonly adopted, and a separation of 4σ between two normal distributions is generally sufficient to prevent overlap between these intervals. As discussed later, the corresponding Bhattacharyya coefficient is 0.134. Accordingly, as a possible benchmark, when the coefficient falls below this value, the probability of misclassifying iron pixels as copper may be considered low. The relationship between distributional separation and the Bhattacharyya coefficient is illustrated in Supplementary Fig. S1 (see Supplementary Materials).

## Results

### CT values

**Fig. 2:**
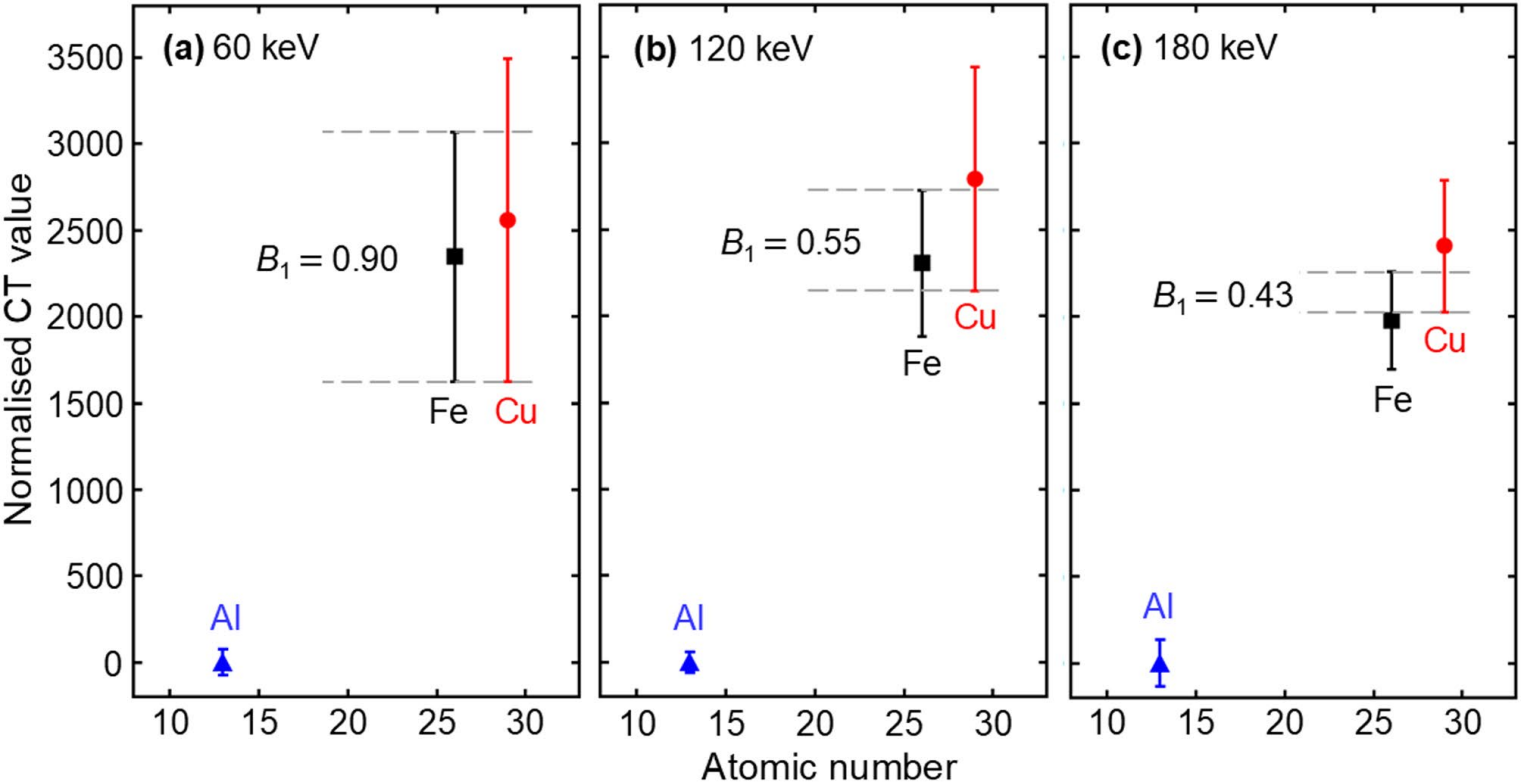
Normalised CT value distributions of the subject cubes (copper, red circle; iron, black square; aluminium, blue triangle) experimentally measured with the STL set at (**a**) 60 keV, (**b**) 120 keV, and (**c**) 180 keV. The normalisation was performed according to Eq. (1), scaling aluminium pixels to 0 and air pixels to −1000 on average. The vertical scale is shared across all three subplots. The error bars indicate ± 2 standard deviations. The horizontal grey dashed lines denote the overlap region between the error bars for iron and copper. The degree of overlap was quantified using the Bhattacharyya coefficient ($0\le{B}_{1}\le1$), where smaller values correspond to greater separation. The formula for calculating the ${B}_{1}$ is Eq. (2a), and the specific calculation examples are provided in Supplementary Materials [see Equations (S1)‒(S5)].

The normalisation described above is based on the observation that the aluminium cube produces minimal beam hardening artefacts, as shown in Fig. 1c. Furthermore, this results in a narrower distribution of the CT values in the aluminium cube compared with those in the other two metal cubes, as shown in Fig. 2. The broader distributions of CT values in iron and copper are significantly caused by artefacts (especially cupping artefacts^40,41^, in which the CT values inside the material decrease while those at the edges are enhanced, as observed in Fig. 1c). Conversely, the decrease and enhancement are almost negligible for the aluminium cube. This normalisation enables quantification of how many times the X-ray attenuation of the materials exceeds that of aluminium. A normalised CT value of 1000 corresponds to twice the attenuation coefficient of aluminium. For example, the normalised CT values (± 2 standard deviations) of the aluminium, iron, and copper cubes are 0 ± 60, 2300 ± 420, and 2790 ± 650, respectively, as shown in Fig. 2b. A known material, such as aluminium, should be used as a reference for normalisation when measuring unknown subjects.

The cupping artefacts caused by beam hardening are apparent in iron and copper cubes in Fig. 1c, enhancing the edges of the subjects^40,41^. This effect is numerically shown in Fig. 1d for the iron cube under condition B. The normalised CT value of the edge pixels (in contact with air) is ca. 2500, gradually decreasing towards the central area to ca. 2000. This decrease leads to broad distributions of CT values for the iron and copper cubes, making these two metals indistinguishable based on their CT values due to overlapping distributions at any STL, as shown in Fig. 2. Specifically, a higher STL tends to result in less overlap and a smaller ${B}_{1}$. However, a substantial overlap persists even at the highest STL of 180 keV, as shown in Fig. 2c. Conversely, discrimination between the period III element (aluminium) and the period IV elements (iron and copper) is not complicated. Medical CT has reported such discrimination between periods, e.g. the discrimination of uric acid stones in the urinary tract consisting only of periods I–II elements (hydrogen, carbon, nitrogen, and oxygen) from calcium stones additionally containing periods III–IV elements (phosphorus, sulphur, and calcium)^42–44^. However, these cases do not involve discrimination between elements, e.g. sulphur (*Z* = 16) versus calcium (*Z* = 20).

### Elemental map

**Fig. 3:**
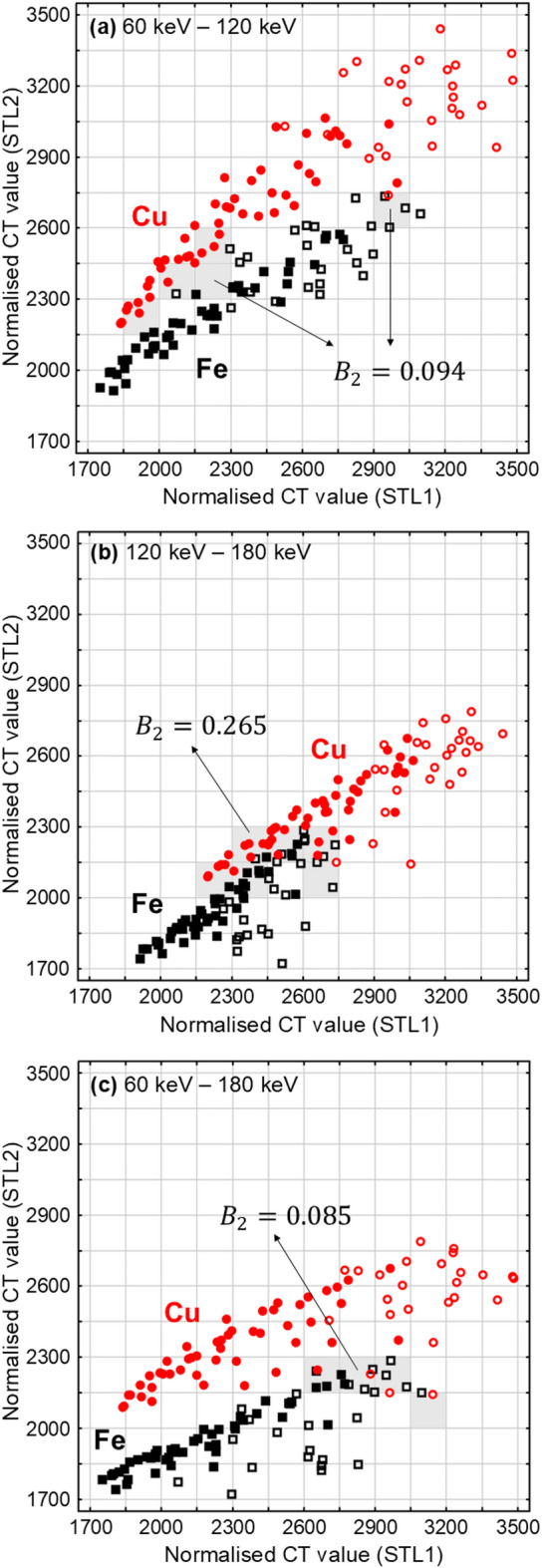
Elemental maps obtained from experimental measurements, with four types of symbols. Red circles denote copper; open circles indicate the outer region of copper (pixels in contact with air), and filled circles indicate the inner region (pixels not in contact with air). Black squares denote iron; open squares indicate the outer region of iron, and filled squares indicate the inner region. Two different STLs are used for the X- and Y-axes, with the combinations of (**a**) 60–120 keV, (**b**) 120–180 keV, and (**c**) 60–180 keV. Both axes are scaled in 150-unit intervals, corresponding to the bin width used for calculating the Bhattacharyya coefficient ($0\le{B}_{2}\le1$). The smaller the value of this metric, the greater the separation between the distributions. Grey hatching indicates the 2D bins in which the copper and iron points coexist; these bins make non-zero contributions in calculating ${B}_{2}$ using Eq. (2b). Specific calculation examples are provided in Supplementary Materials [see Equations (S6)‒(S10)].

The present method for elemental discrimination is sufficiently robust to beam hardening artefacts, and their correction was unnecessary in this study, despite the many correction algorithms previously reported^40,41,45–48^. Instead, our method utilises two STLs. For example, Fig. 3a was generated using two STLs of 60 keV and 120 keV, following the procedure described above. Thus, all the pixels within the iron cube in Fig. 1d are projected with black squares, while those inside the copper cube are projected with red circles. In Fig. 3a and c, the 2D distributions of the black squares and red circles are largely distinct. Consequently, one can map an area on the scatter plot where the likelihood of iron is significantly higher than that of copper, and vice versa. These areas can then be used to estimate the material of a subject (iron or copper, for simplicity) based on which area the majority of its pixels belong. By contrast, Fig. 3b exhibits some overlap between the two symbol types, indicating reduced elemental discriminability when using the two STLs at 120 keV and 180 keV.

### Metric

Normalised CT values of the iron and copper cubes at given STLs were histogrammed, and the frequency vectors were multiplied following Eq. (2a) to obtain the 1D Bhattacharyya coefficients. The calculation results are summarised in Table 2, and the corresponding values are also shown in Fig. 2. As the STL energy increases, the metric decreases, corresponding to improved elemental discriminability. However, a substantial overlap remains even at the highest STL of 180 keV, as shown in Fig. 2c.

**Table 2:** Elemental discriminability by the normalised CT value between iron and copper in Fig. 2, assessed with 1D Bhattacharyya coefficient ($0\le{B}_{1}\le 1$) using Eq. (2a).

STL	60 keV	120 keV	180 keV
${B}_{1}$	0.895	0.548	0.428

Smaller coefficient values indicate reduced overlap between the distributions and thus higher elemental discriminability.

Similarly, the elemental maps at given STL combinations were histogrammed, and the matrices were multiplied following Eq. (2b) to obtain the 2D Bhattacharyya coefficients. The results are summarised in Table 3, and the corresponding values are also shown in Fig. 3. The metrics for the STL combinations of 60–120 keV and 60–180 keV are an order of magnitude smaller than those in the 1D cases, indicating a substantial reduction in overlap. However, the improvement is limited for the STL combination of 120–180 keV, and a considerable overlap persists, as shown in Fig. 3b.

**Table 3:** Elemental discriminability by the elemental map between iron and copper in Fig. 3, assessed with the 2D Bhattacharyya coefficient ($0\le{B}_{2}\le1$) using Eq. (2b).

STL combination	60–120 keV	120–180 keV	60–180 keV
${B}_{2}$	0.094	0.265	0.085

Calculation examples are provided in Supplementary Materials [see Equations (S1)‒(S5) for the 1D case and Equations (S6)‒(S10) for the 2D case].

## Discussion

**Fig. 4:**
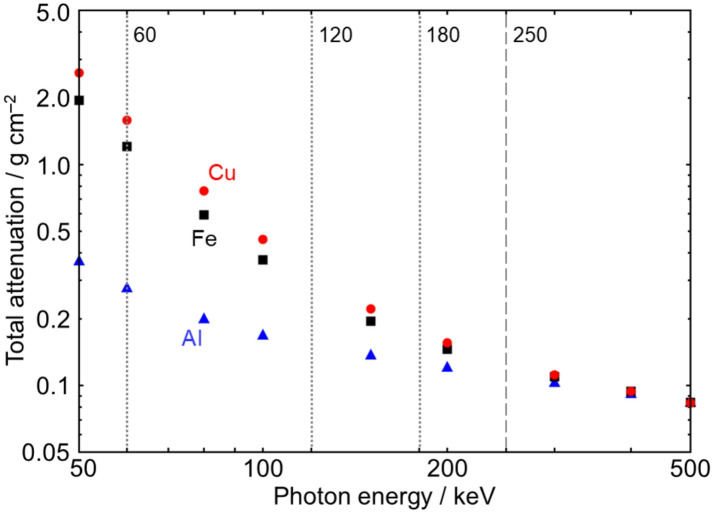
A log-log plot of the mass attenuation coefficient of aluminium (blue triangles), iron (black squares), and copper (red circles) from the database provided by the National Institute of Standards and Technology (NIST) in the United States^12^. The three vertical dotted lines indicate the STLs of 60 keV, 120 keV, and 180 keV, while the vertical dashed line shows the maximum X-ray photon energy (tube voltage) of 250 keV. The separation between the copper and iron lines is larger at lower energies.

Based on these results, improved discriminability can be expected when the two STLs differ more substantially in energy. The STL combination of 60–180 keV, with an energy difference of 120 keV, yields the minimum overlap of ${B}_{2}$ = 0.085. When the energy differences are comparable, the combination of 60–120 keV (${B}_{2}$ = 0.094) performs better than that of 120 keV‒180 keV (${B}_{2}$ = 0.265). This is likely because the difference between the two images is relatively small in the latter case, as the slope of the attenuation coefficient in the 120 keV–180 keV range is lower than that in the 60 keV–120 keV range, as shown in Fig. 4.

The combination of 60 keV‒120 keV is also advantageous in terms of acquisition time, as described in Table 1; this is because the detector sensitivity decreases with increasing STL, as noted above. These advantages contrast with those observed in the 1D cases, where a higher STL consistently results in fewer overlaps between the iron and copper distributions (Fig. 2). These results in the 2D cases indicate that higher STLs do not necessarily improve elemental discrimination, particularly when acquisition time is limited. In each elemental map in Fig. 3, the points are more clustered in the lower-left and more sparse in the upper-right. The upper-right points, corresponding to pixels with relatively high CT values in each subject—primarily edge pixels denoted by open circles and squares—have been affected by cupping artefacts, which degrade the elemental information. Notably, the iron and copper are clearly discriminated in Fig. 3a and c, even without excluding edge pixels from the elemental map or applying a correction algorithm for the artefacts. Furthermore, neither the 60 keV dataset in Fig. 2a nor the 120 keV dataset in Fig. 2b alone enables discrimination; however, their combination achieves it, as shown in Fig. 3a. This improvement demonstrates that the combination enhances elemental information by capturing the element-specific energy dependence of the attenuation coefficient (Fig. 4).

In this way, this method can be applied to smaller subjects (< 20 mm), where the proportion of edge pixels is relatively high, since the elemental map method can handle both outer pixels (open circles and squares in Fig. 3) affected by the cupping effect as well as inner pixels (closed circles and squares). Conversely, for larger subjects, X-ray photons with greater penetrating power (i.e. higher tube voltage) are required to compensate for their increased absorption; this principle applies to elemental discrimination as well as general X-ray CT applications. Our method enhances the elemental information obtained from X-ray attenuation. The amount of 2D information is never less than that of 1D information, since 1D information is a projection of 2D information along a single direction. Specific examples of this projection from 2D to 1D can be found in the Supplementary Material by comparing Equations (S1) and (S6) or Equations (S3) and (S8).

We shall discuss the origin of the elemental information on the elemental map, using a *reductio ad absurdum* argument. Assume that the attenuation coefficient for copper (${\mu}_{\text{Cu}}$) is a constant multiple of that for iron (${\mu}_{\text{Fe}}$), i.e. ${\mu}_{\text{Cu}}\left(E\right)=c{\mu}_{\text{Fe}}\left(E\right)$, where *E* is the energy and *c* is a constant. In this case, the iron and copper points in Fig. 3 would be distributed along the same line, since the CT values are proportional to the attenuation coefficients. For example,

$$\begin{aligned}&{\mu}_{\text{Cu}}(120\,\text{keV}\le E\le250\,\text{keV})/{\mu}_{\text{Cu}}(60\,\text{keV}\le E\le250\,\text{keV})\\ &\quad={\mu}_{\text{Fe}}(120\,\text{keV}\le E\le250\,\text{keV})/{\mu}_{\text{Fe}}(60\,\text{keV}\le E\le 250\,\text{keV})\end{aligned}$$

indicating that the lines for iron and copper would share the same slope. This assertion is contradicted by the actual elemental maps shown in Fig. 3. Therefore, the above assumption is invalid. In other words, the separable distributions of iron and copper along different lines in Fig. 3a and 3c reflect the actual deviations from ${\mu}_{\text{Cu}}\left(E\right)=c{\mu}_{\text{Fe}}\left(E\right)$ in Fig. 4. The elemental maps capture the element-specific energy dependence of the attenuation coefficients.

This consideration should apply to any material composed of multiple elements. By summing the attenuation of each element according to its proportion in the material, one obtains an attenuation curve characteristic of that material^12^. For example, the attenuation coefficients of the urinary stones^42–44^ may exhibit a material-specific energy dependence, allowing the separation of calcium stones from uric acid stones. Even when the conventional CT values of the stones partially overlap, the two-dimensional map may resolve them into two distinct areas, thereby improving diagnostic capability.

Finally, we present the advantages of this method beyond its elemental discriminability. In this study, the three conditions listed in Table 1 were independently measured three times by rotating the table a total of 360° × 3, as the detector had only one STL. However, in principle, our method does not require changing the radiation generator between measurements. Given that the detector is equipped with at least three STLs, the same information can be acquired simultaneously during a 360° rotation. Detectors with these capabilities have been reported elsewhere^11,49^. Therefore, the short measurement time constitutes a fundamental advantage of this method. Another advantage lies in its simplicity. No complex spectral analysis is required; instead, it suffices to perform measurements under two STLs, reconstruct each image individually, and generate the corresponding scatter plot. Moreover, multiple-element discrimination is possible without changing the STL combination, whereas K-edge imaging requires adjusting the tube voltage for each element according to its K-edge energy.

## Conclusion

We have conducted a feasibility study on the elemental discrimination of aluminium, iron, and copper, which belong to periods III–IV of the periodic table and are representative metals in nuclear power plant waste. Our method is characterised by changing the energy threshold level of the pixelated CdTe detector, unlike the conventional techniques, which change the energy spectrum of the radiation generator (e.g. the tube voltage) in accordance with the target element. Aluminium, which produced minimal beam hardening artefacts, was selected as a reference for CT value normalisation. Iron (*Z* = 26) and copper (*Z* = 29) served as a compelling test case for discriminating elements with similar atomic numbers, demonstrating the method’s feasibility.

Beam hardening artefacts prevented discrimination between the elements with a single energy threshold level. By employing two distinct threshold levels, we constructed a 2D scatter plot on which the target elements could be clearly discriminated. We further evaluated elemental discriminability quantitatively using the Bhattacharyya coefficient as a metric. The element discriminability for atomic numbers differing by as little as three may offer further opportunities for photon-counting CT.

Future studies could focus on nickel (*Z* = 28), cupronickel (an alloy of copper and nickel), and brass (an alloy of copper and zinc), which constitute interesting subjects for further investigation. Furthermore, the present findings highlight the method’s robustness against artefacts. At the same time, specific algorithms^40,41,45–48^ and optimised filters^34,35^ can mitigate the artefacts, thereby enabling more accurate discrimination of a broader range of elements. The knowledge gained from this study may serve to assess the activity of radioactive waste from nuclear power plants. Moreover, because our method does not depend on the K-edge energy of the target element, it can be applied broadly to other elements in non-destructive and non-invasive inspections in industrial and biomedical CT contexts.

## Supplementary Information

Below is the link to the electronic supplementary material.

Supplementary Material 1

## Data Availability

The data supporting the findings of this study are available from the corresponding author upon reasonable request.
