# Supplementary material for: Discriminability of selected periods III‒IV elements in photon-counting computed tomography using a pixelated CdTe detector
Source: Sci Rep. 2025 Nov 28;15:42645. doi: 10.1038/s41598-025-26812-5 (PMC12663250; doi:10.1038/s41598-025-26812-5)
Supplement: Supplementary file 1 — Supplementary Material 1 [file 41598_2025_26812_MOESM1_ESM.pdf]

Supplementary Materials

Discriminability of Selected Periods III–IV Elements in Photon-Counting Computed Tomography Using a Pixellated CdTe Detector

Kengo Shibuya[1,*], Shunya Nakasone[1], Taiki Yoshii[1], Akira Yunoki[1], Hirotaka Sakai[1], Hiromi Kimura[2], Takeshi Fujiwara[2], Takeru Takeuchi[3] & Jun Kawarabayashi[3]

[1] Regulatory Standard and Research Department, Nuclear Regulation Authority, Roppongi 1-9-9, Minato-ku, Tokyo 106-8450, Japan

[2] National Metrology Institute of Japan, National Institute of Advanced Industrial Science and Technology, Umezono 1-1-1, Tsukuba, Ibaraki 305-8568, Japan

[3] Faculty of Science and Engineering, Tokyo City University, Tamazutsumi 1-28-1, Setagaya-ku, Tokyo 158-8557, Japan

[*] Corresponding author: shibuya_kengo_e2v@nra.go.jp

Example of metric calculation using actual data (1D)

An example calculation using Equation (2a) is presented for an STL of 120 keV.

Figure 1(c) shows the reconstructed image of the iron cube, in which 72 pixels are identified as being inside the material. Similarly, 74 pixels are identified within the copper cube. The normalised CT values of the two metal cubes range from 1,751 to 3,848. Histograms were constructed by dividing these distributions into twelve discrete intervals between 1,700 and 3,500. Specifically, the first bin spans the range 1,700–1,850, the second 1,850–2,000, and the twelfth 3,350–3,500, with the intermediate bins following the same interval width of 150. The histogram's frequency vector ($\hat{P}$) and normalised frequency vector (P) were obtained by scaling $\hat{P}$ so that the sum of all elements equals one, as follows:

$$\hat{P} = (0,6,14,14,17,13,8,0,0,0,0,0), \quad \text{(S1)}$$

$$P = \hat{P}/72. \quad \text{(S2)}$$

Similarly, the frequency vector for the copper cube ($\hat{Q}$) and the normalised frequency vector (Q) were obtained as follows:

$$\hat{Q} = (0,0,0,6,5,10,13,8,13,7,11,1), \quad \text{(S3)}$$

$$Q = \hat{Q}/74. \quad \text{(S4)}$$

Subsequently, the 1D Bhattacharyya coefficient was calculated using Equation (2a) as follows:

$$B_1 = \sqrt{\frac{14}{72}\times\frac{6}{74}} + \sqrt{\frac{17}{72}\times\frac{5}{74}} + \sqrt{\frac{13}{72}\times\frac{10}{74}} + \sqrt{\frac{8}{72}\times\frac{13}{74}} = 0.5478 \ , \quad \text{(S5)}$$

where terms equal to zero are omitted, as they make no contribution.

Example of metric calculation using actual data (2D)

An example calculation using Equation (2b) is presented for an STL combination of 120 keV–180 keV.

Figure 3(b) shows the elemental map of iron and copper obtained with an STL combination of 120 keV–180 keV. There are 72 iron data points (black squares) and 74 copper data points (red circles). As in the 1D case, normalised CT values were divided into twelve bins, each with a width of 150. The first bin convers the range from 1,700 to 1,850, and the last bin from 3,350 to 3,500. Since there are twelve bins for each STL, the matrix representing the frequency of the 2D histogram ($\hat{P}$) contains 12 × 12 = 144 elements. A normalised frequency matrix (P) was then obtained by scaling $\hat{P}$ so that the sum of all elements equals one, as follows:

$$\hat{P} = \begin{pmatrix} 0 & 0 & 0 & 0 & 0 & 0 & 0 & 0 & 0 & 0 & 0 & 0 \\ 6 & 0 & 0 & 0 & 0 & 0 & 0 & 0 & 0 & 0 & 0 & 0 \\ 4 & 10 & 0 & 0 & 0 & 0 & 0 & 0 & 0 & 0 & 0 & 0 \\ 1 & 12 & 1 & 0 & 0 & 0 & 0 & 0 & 0 & 0 & 0 & 0 \\ 4 & 4 & 7 & 2 & 0 & 0 & 0 & 0 & 0 & 0 & 0 & 0 \\ 2 & 0 & 6 & 5 & 0 & 0 & 0 & 0 & 0 & 0 & 0 & 0 \\ 0 & 1 & 2 & 5 & 0 & 0 & 0 & 0 & 0 & 0 & 0 & 0 \\ 0 & 0 & 0 & 0 & 0 & 0 & 0 & 0 & 0 & 0 & 0 & 0 \\ 0 & 0 & 0 & 0 & 0 & 0 & 0 & 0 & 0 & 0 & 0 & 0 \\ 0 & 0 & 0 & 0 & 0 & 0 & 0 & 0 & 0 & 0 & 0 & 0 \\ 0 & 0 & 0 & 0 & 0 & 0 & 0 & 0 & 0 & 0 & 0 & 0 \\ 0 & 0 & 0 & 0 & 0 & 0 & 0 & 0 & 0 & 0 & 0 & 0 \end{pmatrix}, \tag{S6}$$

$$P = \hat{P}/72. \tag{S7}$$

Note that the vertical axis of the matrix corresponds to an STL of 120 keV, while the horizontal axis corresponds to an STL of 180 keV. Summing the elements in each row yields the above one-dimensional $\hat{P}$.

Similarly, the frequency matrix for the copper cube ($\hat{Q}$) and the normalised frequency matrix (Q) were obtained, as follows:

$$\hat{Q} = \begin{pmatrix} 0 & 0 & 0 & 0 & 0 & 0 & 0 & 0 & 0 & 0 & 0 & 0 \\ 0 & 0 & 0 & 0 & 0 & 0 & 0 & 0 & 0 & 0 & 0 & 0 \\ 0 & 0 & 0 & 0 & 0 & 0 & 0 & 0 & 0 & 0 & 0 & 0 \\ 0 & 0 & 5 & 1 & 0 & 0 & 0 & 0 & 0 & 0 & 0 & 0 \\ 0 & 0 & 1 & 4 & 0 & 0 & 0 & 0 & 0 & 0 & 0 & 0 \\ 0 & 0 & 0 & 8 & 2 & 0 & 0 & 0 & 0 & 0 & 0 & 0 \\ 0 & 0 & 1 & 3 & 8 & 1 & 0 & 0 & 0 & 0 & 0 & 0 \\ 0 & 0 & 0 & 2 & 3 & 3 & 0 & 0 & 0 & 0 & 0 & 0 \\ 0 & 1 & 0 & 0 & 2 & 7 & 3 & 0 & 0 & 0 & 0 & 0 \\ 0 & 0 & 1 & 0 & 0 & 3 & 3 & 0 & 0 & 0 & 0 & 0 \\ 0 & 0 & 0 & 0 & 0 & 2 & 7 & 2 & 0 & 0 & 0 & 0 \\ 0 & 0 & 0 & 0 & 0 & 0 & 1 & 0 & 0 & 0 & 0 & 0 \end{pmatrix}, \tag{S8}$$

$$Q = \hat{Q}/74. \tag{S9}$$

Subsequently, the 2D Bhattacharyya coefficient was calculated using Equation (2b) as follows:

$$B_2 = \sqrt{\frac{5}{72} \times \frac{1}{74}} + \sqrt{\frac{1}{72} \times \frac{7}{74}} + \sqrt{\frac{4}{72} \times \frac{2}{74}} + \sqrt{\frac{8}{72} \times \frac{5}{74}} + \sqrt{\frac{1}{72} \times \frac{2}{74}} + \sqrt{\frac{3}{72} \times \frac{5}{74}} = 0.2647, \tag{S10}$$

where terms equal to zero are omitted, as they make no contribution.

Bhattacharyya coefficient values and distribution overlap

To the best of the authors' knowledge, no universally accepted threshold for the Bhattacharyya coefficient has been established that reliably signifies a substantially low degree of overlap. This is likely because the appropriate value varies according to the field of application and the intended use of the coefficient (e.g. the need for conservative settings). Fig. S1 presents the Bhattacharyya coefficient values for two normal distributions whose means (*i.e.* the positions of the symmetry axes) are separated by n times the standard deviation ($0 \leq n \leq 10$). Thus, when distributions are normally distributed, it is common practice across

many fields to consider a confidence interval spanning two standard deviations (2σ), which encompasses 95.46% of the data. When the distance between the means is 4σ, thereby ensuring these confidence intervals do not overlap, the Bhattacharyya coefficient takes the value $B_1 = 0.134$. As noted in the main text, the CT values reported in this paper are affected by beam hardening and therefore do not conform to a normal distribution. However, as one possible guideline, if $B_1 < 0.134$, we consider the likelihood of misclassifying iron pixels as copper to be low.

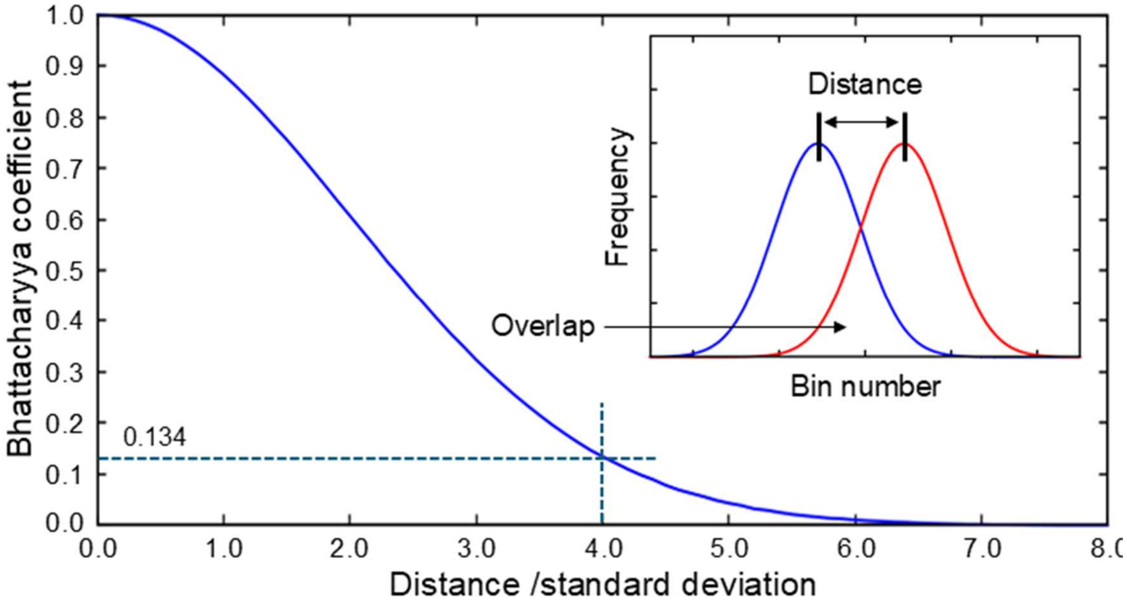

Fig. S1. Relationship between the separation of two normal distributions and the corresponding Bhattacharyya coefficient. The inset illustrates that the separation refers to the distance between the symmetry axes of each distribution, expressed in units of standard deviation. The dotted line indicates that when this distance equals four standard deviations, the Bhattacharyya coefficient is $B_1 = 0.134$
